# Transportability of an artificial intelligence electrocardiography model for structural heart disease: external validation and recalibration of EchoNext-Mini in MIMIC-IV

**DOI:** 10.1093/ehjdh/ztag147

**Published:** 2026-09-19

**Authors:** Emmanuel Otabor, Abdulraheem Hassan, Ayoyimika Okunlola, Justin Lam, Laith Alomari, Michael Hamilton

**Affiliations:** Thomas Jefferson University, Philadelphia, PA 19107, USA; Saint Peter's University Hospital, New Brunswick, NJ, USA; Nottingham University Hospital NHS Trust, Nottingham, UK; Thomas Jefferson University, Philadelphia, PA 19107, USA; Thomas Jefferson University, Philadelphia, PA 19107, USA; Thomas Jefferson University, Philadelphia, PA 19107, USA

**Keywords:** artificial intelligence, Electrocardiography, Echocardiography, Structural heart disease, External validation, Calibration

## Abstract

**Aims:**

Structural heart disease is often underdiagnosed, and echocardiography is limited by access and referral thresholds. Artificial intelligence electrocardiography (AI-ECG) may identify echocardiographically defined structural heart disease. EchoNext-Mini is a released AI-ECG model, accompanied by a benchmark dataset, that detects a composite structural heart disease outcome and component conditions from 12-lead electrocardiogram waveforms together with age, sex, and electrocardiographic measurements. We assessed its transportability and recalibration.

**Methods and results:**

We linked electrocardiograms from MIMIC-IV to subsequent transthoracic echocardiograms, including adults whose electrocardiogram preceded an eligible echocardiogram within one year, one per patient. Labels were mapped to structured echocardiographic fields using original thresholds where available, harmonized definitions otherwise. The released model was applied without retraining to this cohort and its benchmark test set, then assessed for discrimination, calibration, recalibration, subgroups, and label sensitivity. Among 45 878 patients, structural heart disease prevalence was 47.2%. Composite area under the receiver operating characteristic curve was 0.790 (95% confidence interval, 0.786 to 0.794), against 0.820 on the benchmark test set. Component values ranged from 0.679 for left ventricular wall thickness to 0.837 for right ventricular dysfunction. The composite was near-calibrated (slope, 0.93), whereas all component probabilities overestimated risk in both settings. A shift derived from the released training prevalences, requiring no local outcome data, largely corrected it.

**Conclusion:**

EchoNext-Mini transported to MIMIC-IV with composite discrimination within the published external-validation range. Component probabilities behaved as ranking scores rather than absolute risks, a property of the released model rather than the validation setting, and require adjustment before use as risk estimates.

## Graphical Abstract

**Graphical Abstract:**
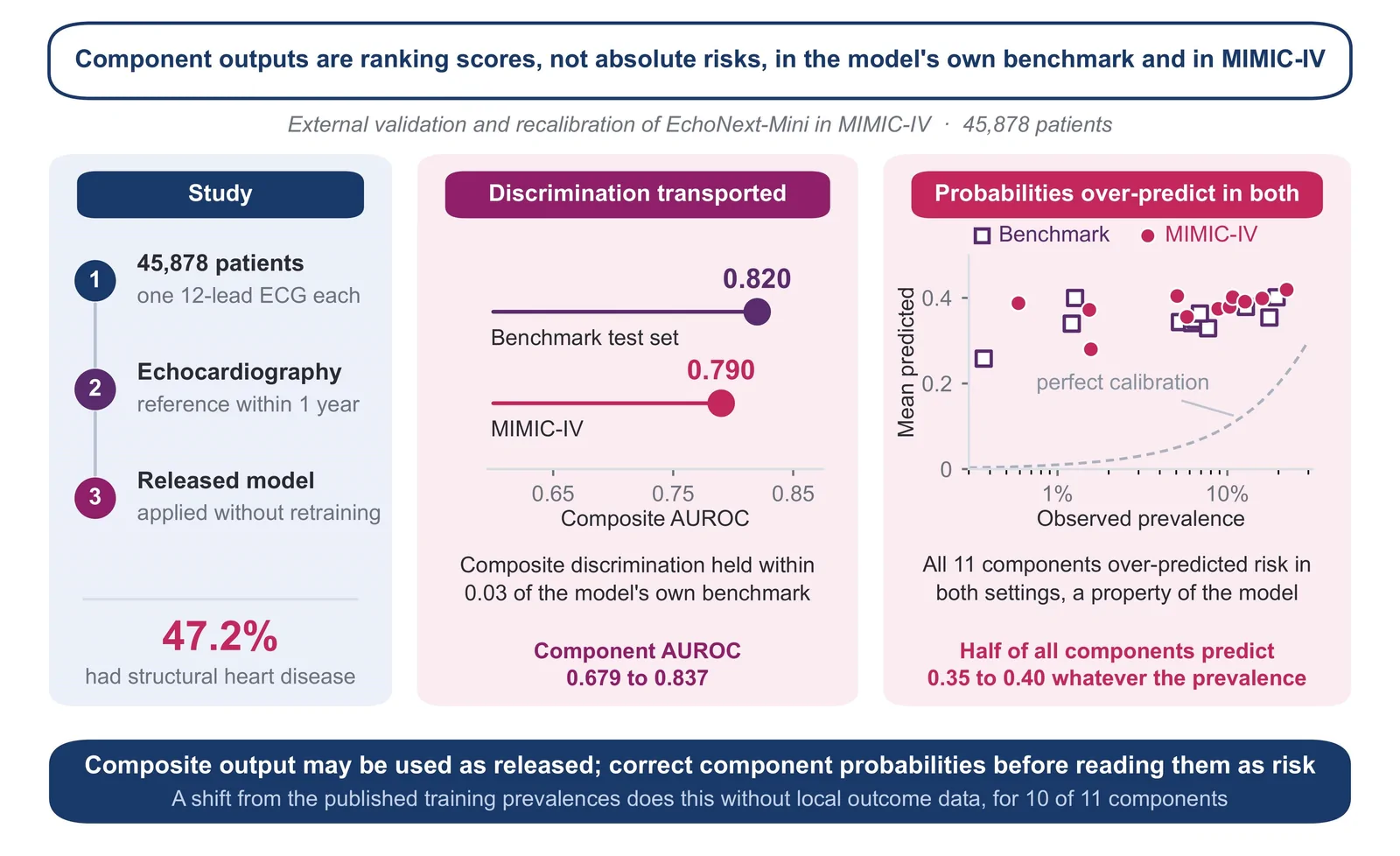

## Introduction

Structural heart disease (SHD) is a broad group of cardiac abnormalities involving valve function, ventricular structure and function, right-sided pressures, pericardial disease, and chamber morphology. Many forms of SHD are treatable but often remain unrecognized until symptoms develop; valvular disease, for instance, is frequently undiagnosed until detected on imaging.^1,2^ Echocardiography is the primary diagnostic test, yet its use is shaped by access, cost, and referral patterns. By contrast, the 12-lead electrocardiogram (ECG) is inexpensive, routinely acquired, and widely available across care settings. These features have made the ECG an attractive substrate for artificial intelligence models that identify patients more likely to have echocardiographically defined disease.

Deep learning applied to ECG waveforms has shown that the electrical signal contains information about occult structural and functional disease, including reduced left ventricular ejection fraction (LVEF), valvular heart disease, and broader SHD composites.^3–8^ EchoNext extended this approach to a multitask model that detects a composite of SHD and its component diagnoses from ECG data. The original EchoNext model was developed using more than one million ECG–echocardiogram pairs from more than 230 000 patients across eight New York-Presbyterian-affiliated hospitals, achieved an internal area under the receiver operating characteristic curve (AUROC) of 85.2%, and was evaluated in additional external health systems and prospective workflows.^9^ EchoNext-Mini was subsequently released, a de-identified benchmark dataset of 100 000 ECGs from NewYork-Presbyterian/Columbia University Irving Medical Center, together with a baseline model that shares the EchoNext architecture and reports an internal composite AUROC of 0.820.^10^ The public release of both the benchmark dataset and baseline model creates an opportunity for independent evaluation in a separate clinical data environment.

Public availability enables reuse, but performance should still be assessed in each new data environment. Transportability can be affected by differences in patient mix, ECG acquisition and preprocessing, label definitions, echocardiography reporting systems, and calibration.^11^ For a model that may support echocardiography referral or prioritization, external evaluation should include calibration, label harmonization, subgroup performance, and reproducibility, rather than discrimination alone. The most closely related prior MIMIC-IV study, TARGET-AI, evaluated a different targeted-deployment framework and used SHD labels extracted from discharge-summary text rather than native structured echocardiographic measurements.^12^ To our knowledge, the released EchoNext-Mini model has not previously been independently validated in MIMIC-IV.

We therefore performed an independent external validation and recalibration of the publicly released EchoNext-Mini model in MIMIC-IV, using structured echocardiographic measurements, verified waveform preprocessing, patient-level evaluation, and sensitivity analyses for label substitutions. We report the study in accordance with the TRIPOD + AI statement.^13^ We frame this work as a test of transportability in a clinically selected population, namely patients with a clinically obtained ECG and a subsequent echocardiogram, rather than as an evaluation of unselected population screening.

## Methods

### Study design and reporting

This was an independent external validation and recalibration study of the publicly released EchoNext-Mini model. The neural-network weights were not retrained; local recalibration was evaluated only as a post-processing step. The model was applied both to the MIMIC-IV cohort and to the released benchmark test set, the latter to verify our implementation and to establish whether calibration behaviour was a property of the model or of the external dataset. Reporting follows the TRIPOD + AI statement for machine-learning prediction models.^13^ The study used de-identified, publicly available data from source databases approved by the Institutional Review Boards of the Beth Israel Deaconess Medical Center and the Massachusetts Institute of Technology, with waiver of individual consent.

### Data sources

We used MIMIC-IV (version 3.1),^14,15^ MIMIC-IV-ECG,^16^ MIMIC-IV-ED,^17^ and MIMIC-IV-ECHO (version 1.0, released 10 March 2026),^18^ all distributed through PhysioNet.^19^ MIMIC-IV is a de-identified, single-centre electronic health record database from the Beth Israel Deaconess Medical Center; MIMIC-IV-ECG provides diagnostic 12-lead waveforms with machine measurements. Labels were derived from the structured echocardiographic measurements of MIMIC-IV-ECHO, which cover 206 488 studies in 91 372 patients.

The released EchoNext-Mini benchmark comprises 100 000 ECGs from 36 286 patients with the published train, validation, and test split; the held-out test set of 5442 ECGs was used as distributed.

### Cohort construction

We included adults (≥18 years) whose 12-lead ECG preceded an eligible transthoracic echocardiogram by up to 365 days, matching the EchoNext labelling window. An eligible echocardiogram reported LVEF and at least one valve finding. Echocardiograms with a repaired or replaced heart valve were excluded, following the original EchoNext methods. ECGs were further excluded for ventricular pacing, poor quality, lead reversal, or invalid measurements. The primary analysis retained one ECG per patient, selected as the most recent eligible recording, with deterministic tie-breaking for reproducibility. Cohort derivation is shown in *Figure 1*.

**Figure 1:**
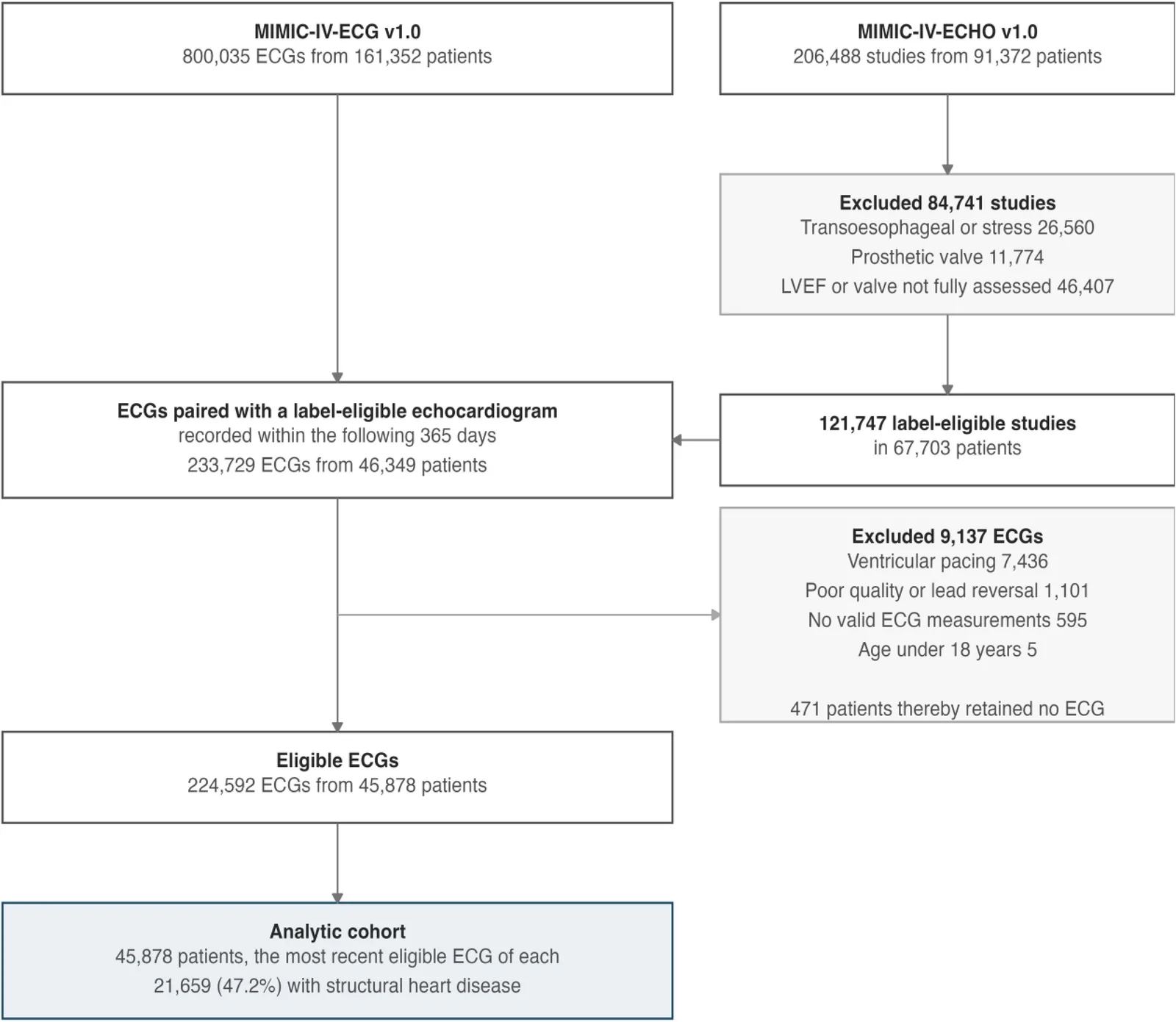
Cohort assembly. Electrocardiograms from MIMIC-IV-ECG were paired with label-eligible transthoracic echocardiograms from MIMIC-IV-ECHO recorded within the following 365 days. Echocardiograms were eligible if they reported left ventricular ejection fraction and at least one valve finding and did not describe a repaired or replaced valve. The analytic cohort retained the most recent eligible ECG for each patient.

Race and ethnicity were assigned from hospital admission and emergency department records. Raw values were grouped into reporting categories before counting, the most frequent informative category was taken in preference to Unknown, and ties were resolved deterministically. Care setting at the time of ECG was assigned by linking the ECG timestamp to concurrent MIMIC-IV intensive care stays, emergency-department visits, and hospital admissions; ECGs without a concurrent encounter were classified as outpatient or ambulatory, except those occurring on the same calendar day as, or the day before, an emergency, urgent, or observation admission, which were classified as acute presentations. Because MIMIC-IV does not provide an explicit outpatient-encounter table, care setting should be interpreted as an encounter-based approximation. An informative value was available only in the emergency department records for 1424 patients.

### Label harmonization

EchoNext labels were mapped to the closest available MIMIC-IV-ECHO structured fields, with original EchoNext thresholds retained where the corresponding structured field was available; full label definitions are provided in Supplementary material online, *Table S1*. When a structured field was absent, the corresponding label was treated as negative, consistent with the EchoNext labelling convention, which was validated in the original report by physician chart review of 50 cases per label, with no more than 1 of 50 cases being misclassified. Field availability varied substantially across labels and is reported with the resulting prevalence under this convention in Supplementary material online, *Table S2*. The SHD composite was positive if any component label was positive.

Several labels required harmonized definitions because MIMIC-IV-ECHO did not encode the EchoNext field directly. Aortic stenosis was taken from the graded severity field where populated, which was the case in 11.5% of studies, and otherwise from guideline quantitative thresholds (peak velocity ≥3.0 m/s, mean gradient ≥20 mmHg, or valve area ≤1.5 cm^2^),^20^ which raised ascertainment to 92.5%, showing that 278 of 40 606 previously assumed negatives (0.68%) were in fact positive; velocities or gradients attributed to high flow rather than stenosis were not counted. The original EchoNext rule also used peak aortic velocity. TR velocity was taken from the structured velocity field, with the equivalent pressure-gradient threshold evaluated in sensitivity analysis. PASP was reconstructed from the TR gradient plus RAP estimated from the inferior vena cava using American Society of Echocardiography categories.^21^ LV wall thickness was approximated as the maximum of septal and inferolateral wall thickness because MIMIC-IV-ECHO records no posterior wall measurement. RV dysfunction was defined primarily from categorical RV function descriptors, with TAPSE evaluated as a sensitivity definition.

### Model implementation and preprocessing

We applied the released EchoNext-Mini weights without retraining.^10^ Waveforms were read from Waveform Database records, reordered to the model lead order, downsampled from 500 to 250 Hz, baseline-wander corrected, clipped, normalized using the released per-lead parameters, and shaped to the model input. Seven tabular features were supplied: sex, age, ventricular rate, atrial rate, PR interval, QRS duration, and corrected QT interval. MIMIC-IV-ECG distributes fiducial points rather than derived intervals, so the intervals were computed from those points and QT was corrected using Bazett's formula, the default of the recording platform on which the model was developed. Following the released preprocessing specification, atrial rate and PR interval were assigned a value of zero before scaling when unavailable, rather than imputed. Atrial rate is not reported in MIMIC-IV-ECG; PR interval was unmeasurable in 15.3% of the cohort, most often in atrial fibrillation. All ECGs in the final cohort were readable 12-lead, 10-second recordings.

Implementation was verified at two levels. Feature construction and inference reproduced the official released module exactly, to a maximum absolute difference of zero across all twelve outputs, on a test set stratified to include missing and extreme measurements (see Supplementary material online, *Table S3*). Applying the same frozen weights to the released benchmark test set reproduced the per-label discrimination published for that model.

### Statistical analysis

Discrimination was assessed using AUROC and the area under the precision-recall curve (AUPRC), the latter also expressed relative to prevalence as (AUPRC − prevalence)/(1 − prevalence). Calibration was assessed using mean predicted probability, calibration-in-the-large (observed prevalence minus mean predicted probability, on the probability scale), the calibration slope from unpenalized logistic recalibration of the outcome on the predicted logit, the Brier score, the Brier skill score relative to a predictor assigning the observed prevalence to every patient,^22^ and quantile-binned reliability curves. Confidence intervals were calculated by bootstrap resampling at patient level, with 2000 resamples and a fixed seed, and are reported as percentile intervals. The same resample indices were used for every label and subgroup. Bootstrap resamples containing no events or no non-events for the label being evaluated were discarded.

Two recalibration strategies were compared: a closed-form prior shift, adding the logit of the label’s prevalence in the released training split to the predicted logit, which requires no outcome data from the target setting;^23,24^ and five-fold out-of-fold Platt scaling fitted separately for each label, which does. The training prevalences were taken from the released benchmark metadata file and are therefore available to any user of the release.

Subgroup performance was assessed by sex, age group, race or ethnicity, care setting, and ECG acquisition settings (recording bandwidth and filtering). Sensitivity analyses evaluated ECG-to-echocardiogram linkage windows, LVEF definitions, RAP assumptions, TR velocity definitions, RV dysfunction definitions, the aortic stenosis definition, the atrial-rate fill rule, exclusion of studies in which RV function was recorded as unassessable, and, for every label with incomplete field availability, restriction to studies in which the field was populated. Because the primary analysis used one ECG per patient, bootstrap resampling and cross-validation folds were patient-level.

In additional analyses, the composite score was evaluated as a prioritization tool under fixed echocardiographic capacity, adapting the design of a recent capacity-constrained triage study,^25^ and by decision-curve analysis of net benefit across threshold probabilities.^26^ Because MIMIC-IV does not record a referral sequence, the unprioritized comparator was a random ordering of the same cohort. As a secondary analysis, the wall-thickness probability was evaluated against two higher-grade hypertrophy outcomes, septal thickness ≥1.5 cm and categorical moderate-or-severe left ventricular hypertrophy.

Analyses were performed with a single analysis module reading one label source, so that all reported quantities derive from the same cohort definition. The authors used AI-assisted tools to support code review; all analyses, results, and final text were reviewed and verified by the authors.

## Results

### Cohort

The primary cohort included 45 878 patients, each contributing one ECG, with a median age of 68 years (IQR, 56–79); 48.6% were female. By care setting, ECGs were obtained in the inpatient ward (31.2%), emergency or acute presentations (28.8%), outpatient or ambulatory settings (24.4%), and intensive care (15.6%). SHD was present in 47.2%, and the median ECG-to-echocardiogram interval was two days. Compared with the patient-level EchoNext-Mini benchmark cohort, MIMIC-IV patients were older, more often ambulatory, and differed markedly in race and ethnicity, while overall prevalence was broadly similar. Cohort characteristics are given in *Table 1*; cohort derivation is shown in *Figure 1*.

**Table 1:** Cohort characteristics of the MIMIC-IV external-validation cohort and the EchoNext-Mini benchmark cohort

Characteristic	MIMIC-IV (this study)	EchoNext-Mini, latest ECG per patient (published)
Patients, *n*	45 878	36 286
Age, years, median [IQR]	68 [56–79]	64 [52–75]
Female, %	48.6	50.5
**Race or ethnicity, %**		
White	69.3	29.3
Black	13.2	14.9
Hispanic	4.6	30.3
Asian	3.2	3.0
Other or unknown	9.7	22.4
**Care setting at ECG, %**		
Inpatient ward	31.2	41.2
Emergency or acute	28.8	35.9
Outpatient or ambulatory	24.4	18.8
Intensive care	15.6	Not reported separately
**Electrocardiographic measurements, median**		
Ventricular rate, bpm	76	78
PR interval, ms	158	156
QRS duration, ms	93	88
QTc, ms	444	444
PR interval unmeasurable, %	15.3	11.2
**Structural heart disease prevalence, %**		
Any (composite)	47.2	43.3
Reduced LVEF	16.0	18.0
LV wall thickness	22.4	18.8
RV dysfunction	5.8	8.0
Pericardial effusion	0.6	1.3
Elevated PASP	10.8	12.9
Elevated TR *V*_max_	12.7	6.5
Aortic stenosis	5.1	5.4
Aortic regurgitation	1.5	1.2
Mitral regurgitation	8.8	6.5
Tricuspid regurgitation	10.3	7.0
Pulmonic regurgitation	1.6	0.4
**ECG-to-echocardiogram interval, days, median [IQR]**	2 [1–15]	not available

Values are shown at the patient level using one ECG per patient. The EchoNext-Mini column reproduces the published values of the Latest ECG column of *Table 1* in Hughes *et al*. and was not recalculated here.^10^

ECG, electrocardiogram; IQR, interquartile range; LVEF, left ventricular ejection fraction; LV, left ventricular; PASP, pulmonary artery systolic pressure; RV, right ventricular; TR, tricuspid regurgitation.

### Discrimination, calibration, and recalibration

For the composite, EchoNext-Mini achieved an AUROC of 0.790 (95% CI, 0.786–0.794) and an AUPRC of 0.772, against a no-skill value of 0.472 at the observed prevalence, giving a prevalence-normalized AUPRC of 0.568. Discrimination was strongest for RV dysfunction and reduced LVEF and weakest for LV wall thickness. Full per-label discrimination and calibration are shown in *Table 2*, and ROC curves for the composite and strongest components in *Figure 2*.

**Figure 2:**
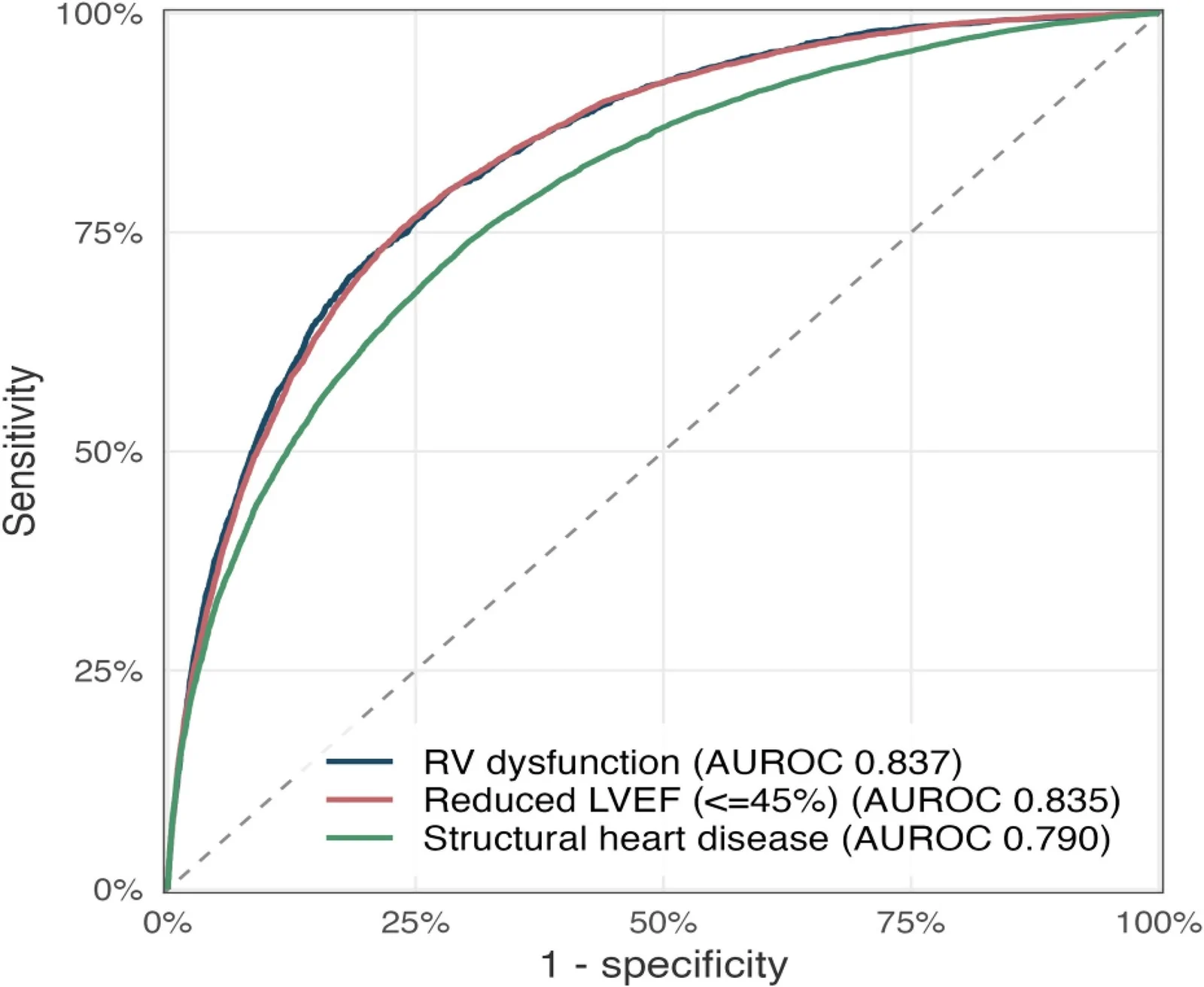
Receiver operating characteristic curves. Curves are shown for the composite structural heart disease label and the two strongest component labels, with areas under the curve given in the legend.

**Table 2:** Discrimination and calibration by label in MIMIC-IV

Label	Prevalence, %	Mean predicted probability	AUROC (95% CI)	AUPRC	Normalized AUPRC	CIL	Calibration slope	Brier	Brier skill
Reduced LVEF (≤45%)	16.0	0.399	0.835 (0.830–0.839)	0.512	0.419	−0.238	1.15	0.175	−0.301
LV wall thickness (≥1.3 cm)	22.4	0.419	0.679 (0.673–0.684)	0.349	0.162	−0.195	0.79	0.207	−0.191
Aortic stenosis	5.1	0.404	0.783 (0.775–0.792)	0.144	0.098	−0.354	0.94	0.215	−3.464
Aortic regurgitation	1.5	0.372	0.730 (0.713–0.746)	0.039	0.024	−0.357	0.83	0.191	−11.595
Mitral regurgitation	8.8	0.374	0.806 (0.799–0.812)	0.286	0.217	−0.286	1.05	0.180	−1.239
Tricuspid regurgitation	10.3	0.379	0.805 (0.799–0.812)	0.330	0.253	−0.276	1.09	0.171	−0.852
Pulmonic regurgitation	1.6	0.280	0.723 (0.703–0.743)	0.051	0.036	−0.264	0.56	0.137	−7.870
RV dysfunction	5.8	0.356	0.837 (0.829–0.844)	0.282	0.238	−0.298	1.11	0.168	−2.076
Pericardial effusion	0.6	0.388	0.765 (0.739–0.792)	0.019	0.013	−0.382	1.36	0.175	−28.960
Elevated PASP (≥45 mmHg)	10.8	0.402	0.768 (0.762–0.774)	0.270	0.182	−0.294	1.09	0.188	−0.961
Elevated TR *V*_max_ (≥3.2 m/s)	12.7	0.391	0.763 (0.757–0.769)	0.301	0.200	−0.264	1.04	0.183	−0.646
Structural heart disease	47.2	0.444	0.790 (0.786–0.794)	0.772	0.568	+0.028	0.93	0.187	+0.249

CIL is observed prevalence minus mean predicted probability. Normalized AUPRC is (AUPRC − prevalence)/(1 − prevalence). Brier skill is 1 − Brier/[*p*(1 − *p*)]; negative values indicate performance worse than assigning the observed prevalence to every patient. Intervals are percentile bootstrap, 2000 resamples, seed 0.

AUPRC, area under the precision-recall curve; AUROC, area under the receiver operating characteristic curve; CIL, calibration-in-the-large; LV, left ventricular; PASP, pulmonary artery systolic pressure; RV, right ventricular; TR, tricuspid regurgitation.

The released model was also applied to its own benchmark test set (*n* = 5442). Per-label AUROC reproduced the published values exactly for ten of twelve outputs, the exceptions being the two labels with fewest events in that test set. Composite AUROC was 0.820 on the benchmark against 0.790 in MIMIC-IV, and five of eleven components transported within 0.010 (*Table 3*).

**Table 3:** The released model applied in two settings: its own benchmark test set and MIMIC-IV

Label	Benchmark					MIMIC-IV				AUROC difference
	Prevalence, %	AUROC (this study)	AUROC (as published)	Mean predicted	CIL	Prevalence, %	AUROC	Mean predicted	CIL	
Reduced LVEF	17.7	0.852	0.852	0.354	−0.177	16.0	0.835	0.399	−0.238	−0.017
LV wall thickness	19.5	0.734	0.734	0.401	−0.206	22.4	0.679	0.419	−0.195	−0.056
Aortic stenosis	5.3	0.859	0.859	0.343	−0.291	5.1	0.783	0.404	−0.354	−0.076
Aortic regurgitation	1.2	0.740	0.739	0.340	−0.328	1.5	0.730	0.372	−0.357	−0.010
Mitral regurgitation	6.2	0.806	0.806	0.341	−0.279	8.8	0.806	0.374	−0.286	−0.000
Tricuspid regurgitation	6.5	0.833	0.833	0.349	−0.284	10.3	0.805	0.379	−0.276	−0.028
Pulmonic regurgitation	0.4	0.832	0.829	0.258	−0.254	1.6	0.723	0.280	−0.264	−0.108
RV dysfunction	7.7	0.866	0.866	0.329	−0.252	5.8	0.837	0.356	−0.298	−0.029
Pericardial effusion	1.3	0.766	0.766	0.400	−0.387	0.6	0.765	0.388	−0.382	−0.001
Elevated PASP	12.8	0.770	0.770	0.379	−0.251	10.8	0.768	0.402	−0.294	−0.002
Elevated TR *V*_max_	6.9	0.754	0.754	0.363	−0.295	12.7	0.763	0.391	−0.264	+0.010
SHD (composite)	42.6	0.820	0.820	0.412	+0.014	47.2	0.790	0.444	+0.028	−0.029

Benchmark AUROC (this study) was computed from the released arrays as distributed; benchmark AUROC (as published) is Supplementary material online, *Table S15* of Poterucha *et al*.^9^ Mean predicted probability and CIL are shown for both settings. Labels are defined as in Supplementary material online, *Table S1*: reduced LVEF ≤45%; LV wall thickness ≥1.3 cm; PASP ≥45 mmHg; TR *V*_max_ ≥3.2 m/s; valvular lesions and pericardial effusion moderate or greater.

AUROC, area under the receiver operating characteristic curve; CIL, calibration-in-the-large; LVEF, left ventricular ejection fraction; LV, left ventricular; PASP, pulmonary artery systolic pressure; RV, right ventricular; SHD, structural heart disease; TR, tricuspid regurgitation.

The composite was near-calibrated in both settings (calibration-in-the-large +0.014 and +0.028 respectively). Component probabilities behaved differently: mean predicted probability lay between 0.28 and 0.42 in MIMIC-IV and between 0.26 and 0.40 on the benchmark, despite prevalences of 0.6% to 22.4% and 0.4% to 19.5% respectively. Brier skill was negative for all eleven components in MIMIC-IV and for ten of eleven on the benchmark, indicating accuracy below that obtained by assigning the observed prevalence to every patient, and positive for the composite in both (*Table 2*; Supplementary material online, *Table S4*). Reliability curves are shown in *Figure 3* and Supplementary material online, *Figure S1*.

**Figure 3:**
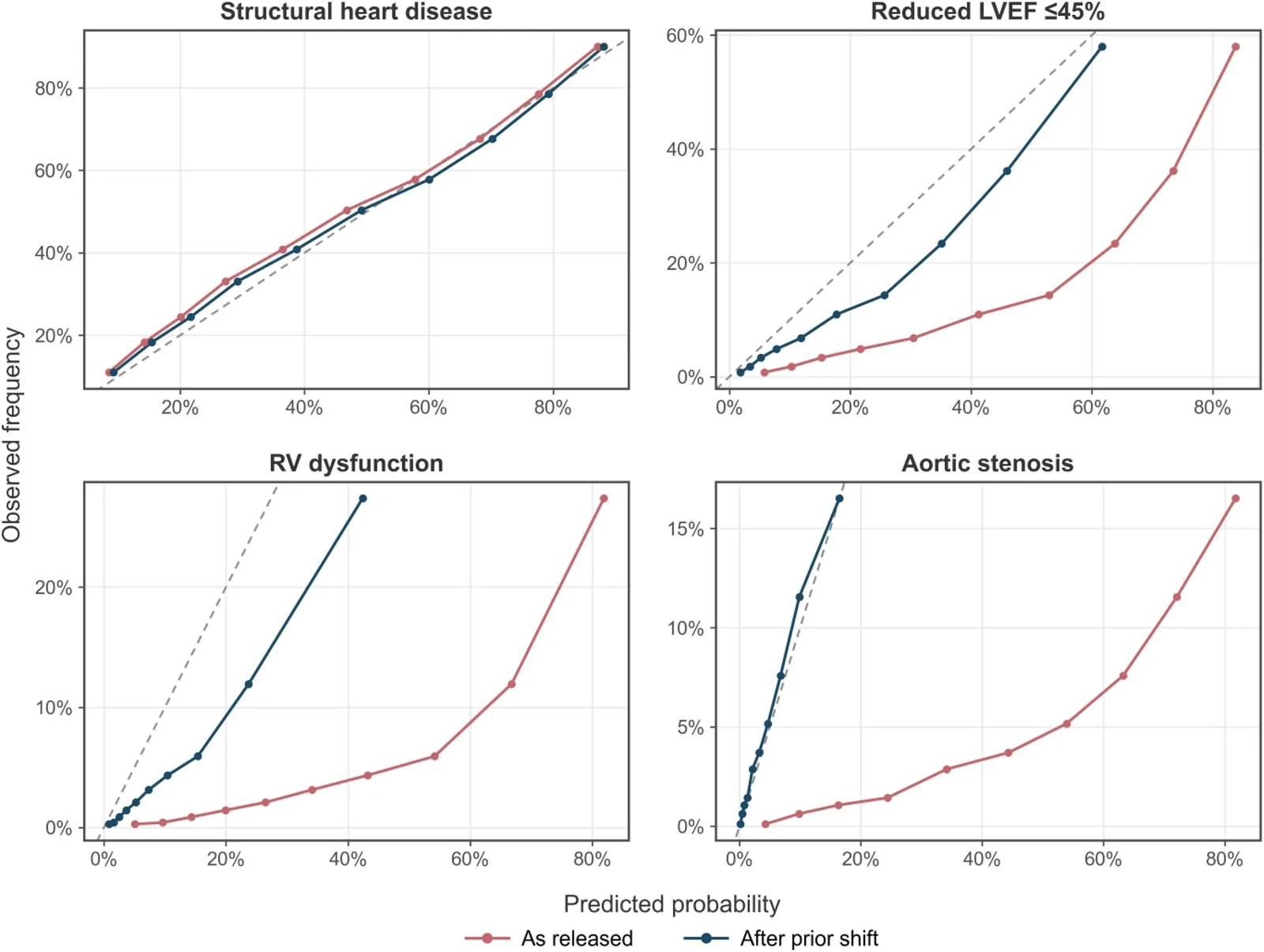
Reliability curves before and after correction. Observed frequency against predicted probability in equal-sized bins, for the composite label and three representative components, as released and after the prior-shift correction. Panel scales differ because prevalence ranges from 5.1% to 47.2% across these four labels.

Two recalibration strategies were compared (*Table 4*). A closed-form prior shift, which adds the logit of the released training prevalence and requires no local outcome data, reduced mean absolute calibration-in-the-large on the benchmark from 0.251 to 0.014 across the twelve outputs and produced positive Brier skill for every component. Five-fold out-of-fold Platt scaling, which requires local labels, performed similarly for most labels and better where local prevalence diverged furthest from training prevalence, most notably pericardial effusion. Calibration slopes were essentially unchanged by the prior shift, as expected of an intercept correction.

**Table 4:** Effect of two recalibration strategies in MIMIC-IV

Label	Prevalence, %	As released			Prior shift			Local Platt		
		CIL	Slope	Brier skill	CIL	Slope	Brier skill	CIL	Slope	Brier skill
Reduced LVEF (≤45%)	16.0	−0.238	1.15	−0.301	−0.056	1.15	+0.202	−0.000	1.00	+0.235
LV wall thickness (≥1.3 cm)	22.4	−0.195	0.79	−0.191	+0.013	0.79	+0.063	+0.000	1.00	+0.066
Aortic stenosis	5.1	−0.354	0.94	−3.464	+0.005	0.94	+0.054	−0.000	1.00	+0.054
Aortic regurgitation	1.5	−0.357	0.83	−11.595	+0.005	0.87	+0.009	+0.000	0.95	+0.011
Mitral regurgitation	8.8	−0.286	1.05	−1.239	+0.009	1.05	+0.117	−0.000	1.00	+0.120
Tricuspid regurgitation	10.3	−0.276	1.09	−0.852	+0.008	1.10	+0.138	−0.000	1.00	+0.141
Pulmonic regurgitation	1.6	−0.264	0.56	−7.870	+0.010	0.56	+0.008	+0.000	0.99	+0.015
RV dysfunction	5.8	−0.298	1.11	−2.076	−0.055	1.11	+0.038	+0.000	1.00	+0.135
Pericardial effusion	0.6	−0.382	1.36	−28.960	−0.016	1.30	−0.059	−0.000	0.97	+0.006
Elevated PASP (≥45 mmHg)	10.8	−0.294	1.09	−0.961	−0.057	1.09	+0.052	+0.000	1.00	+0.095
Elevated TR *V*_max_ (≥3.2 m/s)	12.7	−0.264	1.04	−0.646	+0.036	1.04	+0.084	−0.000	1.00	+0.103
Structural heart disease	47.2	+0.028	0.93	+0.249	+0.011	0.93	+0.252	+0.000	1.00	+0.253

The prior shift adds the logit of the label's prevalence in the released training split to the predicted logit and uses no outcome data from this cohort. Platt scaling is five-fold out-of-fold logistic recalibration fitted on this cohort. Slopes are shown for both.

CIL, calibration-in-the-large; LVEF, left ventricular ejection fraction; LV, left ventricular; PASP, pulmonary artery systolic pressure; RV, right ventricular; TR, tricuspid regurgitation.

### Subgroup performance

Composite AUROC showed no large differences by sex or reported race and ethnicity, ranging from 0.778 to 0.806, but decreased with age, from 0.773 below 65 years to 0.732 at 80 years or older, alongside rising SHD prevalence across the same strata (*Table 5*). Discrimination was likewise lower among inpatients and intensive care patients (0.773) than outpatients (0.796). Performance by ECG acquisition setting is given in Supplementary material online, *Table S5* and Supplementary material online, *Figure S2*.

**Table 5:** Subgroup discrimination and calibration for the composite label

Subgroup	*n*	Prevalence, %	AUROC (95% CI)	Calibration slope
**Sex**				
Male	23 569	51.4	0.778 (0.772–0.784)	0.89
Female	22 309	42.7	0.801 (0.795–0.806)	0.97
**Age**				
Under 65 years	19 492	31.7	0.773 (0.766–0.779)	0.91
65–79 years	15 360	50.9	0.751 (0.743–0.758)	0.84
80 years and older	11 026	69.5	0.732 (0.722–0.742)	0.79
**Race or ethnicity**				
White	31 775	48.3	0.786 (0.781–0.791)	0.93
Black	6053	47.9	0.802 (0.791–0.812)	0.97
Hispanic	2116	37.9	0.778 (0.758–0.799)	0.89
Asian	1486	38.4	0.806 (0.783–0.830)	0.98
Other	1706	40.7	0.802 (0.781–0.823)	0.97
Unknown	2742	49.1	0.798 (0.781–0.814)	0.94
**Care setting at ECG**				
Emergency or acute presentation	13 191	44.4	0.785 (0.778–0.793)	0.93
Inpatient or intensive care	21 492	54.3	0.773 (0.767–0.780)	0.90
Outpatient or ambulatory	11 195	36.9	0.796 (0.787–0.805)	0.99

Race and ethnicity are assigned from both hospital admission and emergency department records. Prevalence differs markedly across strata and should be read alongside discrimination. Intervals are percentile bootstrap, 2000 resamples, seed 0.

AUROC, area under the receiver operating characteristic curve; CI, confidence interval; ECG, electrocardiogram.

### Prioritization of echocardiography

Because ranking is unaffected by the calibration offset, the composite was evaluated as a prioritization tool under fixed echocardiographic capacity. Where capacity permitted imaging 10% of the cohort, ranking by model score identified 19.1% of all SHD compared with 10.0% under unprioritized referral, and 90.1% of studies performed were positive; identifying half of all cases required imaging 30.0% of the cohort rather than 50.0%. Net benefit was comparable to imaging all patients at low threshold values and exceeded it from approximately 0.10 upward (*Figure 4*; Supplementary material online, *Table S6*). The comparator is a random ordering of the same queue, and at the observed prevalence the maximum achievable enrichment is 2.12.

**Figure 4:**
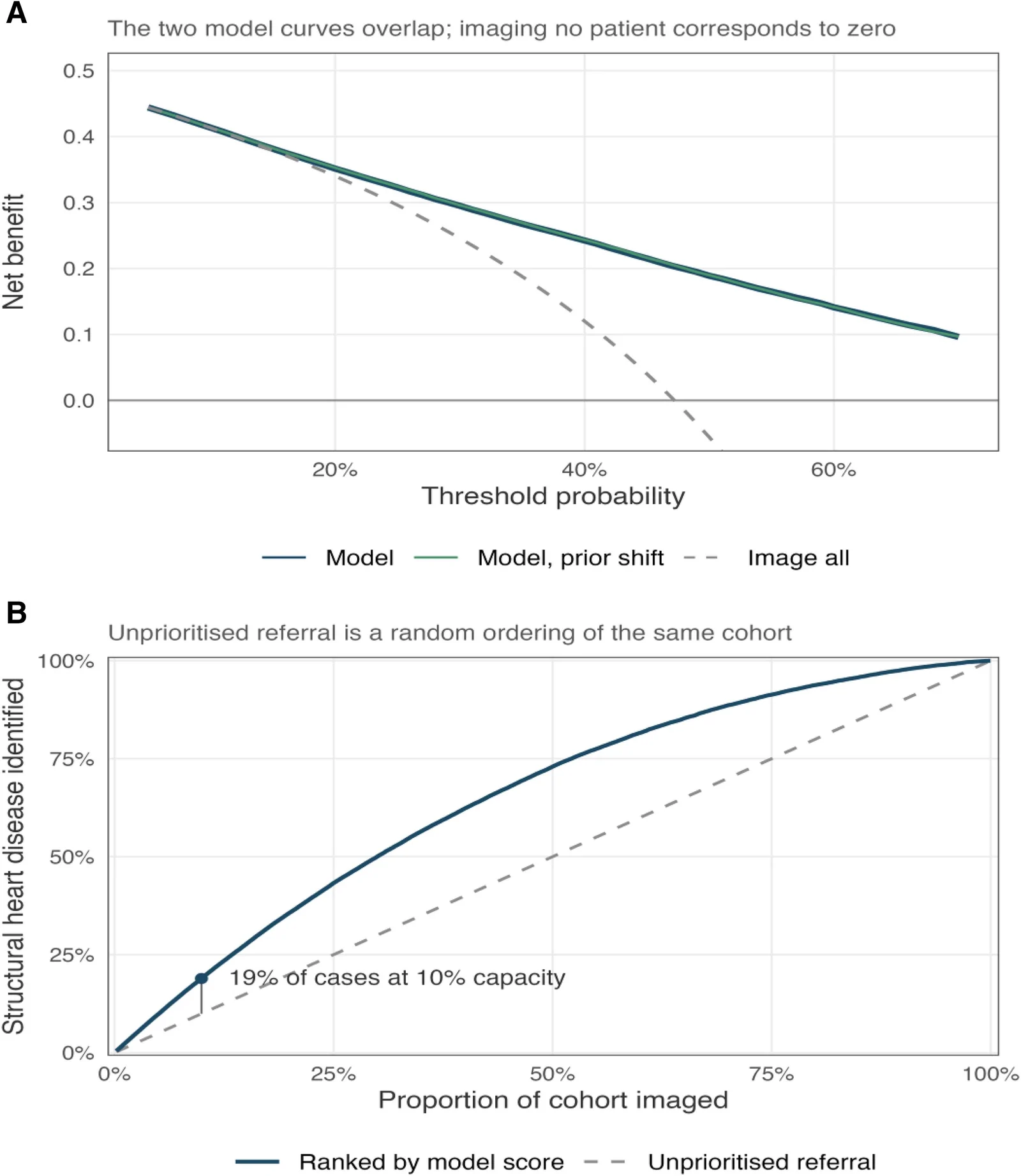
Clinical utility of the composite output. (*A*) Net benefit against threshold probability for the model, the model after prior-shift correction, and imaging every patient; the two model curves overlap because the composite is already calibrated. (*B*) Cumulative diagnostic yield against the proportion of the cohort imaged, for ranking by model score and for unprioritized referral.

### Sensitivity analyses

Discrimination for two labels was sensitive to the treatment of ungraded fields as negative (see Supplementary material online, *Table S7*). Restricting to studies in which the field was populated reduced AUROC from 0.730 to 0.634 for aortic regurgitation and from 0.765 to 0.680 for pericardial effusion, the two least completely graded labels. The two estimates are biased in opposite directions and are best read as bounds. Labels with near-complete ascertainment were unchanged, including aortic stenosis (0.783 to 0.782) and mitral regurgitation (0.806 to 0.806).

Findings were otherwise stable. Composite AUROC remained approximately 0.79 across ECG-to-echocardiogram windows of 30–365 days, and component analyses spanning the LVEF, RV, TR velocity, PASP, and wall-thickness definitions showed similar discrimination and calibration across alternatives, as did exclusion of the 1090 studies in which RV function was recorded as unassessable, and median imputation of atrial rate in place of the released zero-fill rule, which left discrimination unchanged for every label and altered calibration-in-the-large by no more than 0.03. Per-variant results are reported in Supplementary material online, *Table S8*.

## Discussion

In this independent validation of the publicly released EchoNext-Mini model, the model showed moderate-to-good transportability in MIMIC-IV, with preserved discrimination and near-baseline calibration for the composite SHD label. The composite AUROC of 0.790 fell within the 0.78–0.80 external-validation range reported for the original EchoNext model.^9^ This performance was observed in a different health system and clinical data environment: EchoNext-Mini was trained on a single-centre subset from NewYork-Presbyterian/Columbia University Irving Medical Center, whereas this study evaluated the released model in Beth Israel Deaconess Medical Center. Although overall patient-level SHD prevalence was broadly similar between MIMIC-IV and the patient-level EchoNext-Mini subset, the cohorts differed in age, race and ethnicity distribution, and component-label prevalence. Calibration behaved differently for the composite label and for its eleven components, and that distinction governs how the model can be used.

This study addresses a different question from the original EchoNext studies, which established the model family across a large health system and prospective workflows and then released a public benchmark and baseline model.^9,10^ Ours tested whether an independent group could apply the released model to a separate public database with reproducible preprocessing, structured-label mapping, calibration assessment, recalibration, and subgroup evaluation. Applied to the benchmark test set distributed with the model, the released weights reproduced the published per-label discrimination almost exactly (*Table 3*), so differences observed in MIMIC-IV reflect the new setting rather than the inference pipeline used here. Public release of model weights enables reuse, but does not by itself establish transportability, calibration, or label fidelity in a new data environment.

Calibration separated the composite from its components. The composite label was near-calibrated at baseline. All eleven component probabilities overestimated risk, and did so on the benchmark test set as well as in MIMIC-IV, so the overestimation is a property of the released model rather than of the validation setting. The trained output layer points the same way: the twelve bias terms all lie within ±0.05, although the conditions they predict differ in frequency by more than sixtyfold in the training data, whereas outputs on a prevalence scale would carry a bias close to the log-odds of each label’s training prevalence (see Supplementary material online, *Figure S3*). This pattern, in which discrimination transports better than absolute-risk calibration, is well described,^27,28^ though here it originates in the released model rather than in differences of case mix. EchoNext-Mini scores may therefore be more reliable for ranking or triage than for estimating component-level risk without local recalibration.

Two corrections were compared. Out-of-fold Platt scaling, which requires local outcome labels, restored positive Brier skill for every component (*Table 4*). Because the miscalibration is a constant offset rather than a distortion of ranking, it can also be corrected from the label frequencies of the released training split, without any local echocardiographic data,^23,24^ which matters for services that cannot assemble local labels. Ranking is likewise unaffected by a constant shift in score, and the component-level offset does not affect the composite output, which showed near-baseline calibration in both settings. This composite is the output relevant to the two uses that Poterucha *et al*. distinguish: a safety-net use, in which the model prompts an additional echocardiogram for a patient who would not otherwise be referred, and a gatekeeper use, in which it informs whether a patient proceeds to echocardiography at all.^9^ In this cohort, every patient had already been referred for and received echocardiography. The analysis therefore examines prioritization among these patients under a hypothetical capacity limit, rather than identifying additional patients for referral through a safety-net approach. Ordering by model score concentrated disease into the studies performed earliest (*Figure 4*), although almost half the cohort had SHD, so the gain available from reordering is bounded. Because this is a referred rather than a screening population, the capacity-constrained analysis projects potential gains from prioritization where echocardiographic capacity is limited; it does not demonstrate those gains in that setting.

Component performance varied in a clinically interpretable pattern. Reduced LVEF and RV dysfunction were among the strongest labels, consistent with prior work showing detectable ECG signatures of ventricular dysfunction.^3,4^ LV wall thickness was the weakest label, as it was in EchoNext-Mini, and this is consistent with the limited sensitivity of the ECG for mild hypertrophy.^10^ Secondary analyses showed stronger discrimination for higher-grade hypertrophy definitions, supporting the interpretation that the weaker result reflects the difficulty of detecting mild wall thickening rather than failure of the broader construct. Two further explanations were examined and neither was supported. Incomplete grading does not account for the aortic stenosis result, since restricting the analysis to graded studies left discrimination unchanged (see Supplementary material online, *Table S7*). The absent posterior-wall measurement does not account for the wall-thickness result either, since omitting either wall in the benchmark, where both are recorded, altered discrimination by no more than 0.014 (see Supplementary material online, *Table S9*).

Label harmonization was central to interpreting component-level performance. Some labels mapped directly to MIMIC-IV-ECHO structured fields, whereas others required harmonized approximations, including reconstructed PASP, LV wall-thickness approximation, and categorical RV dysfunction definitions. The stability of findings across alternative definitions supports the robustness of the main results, but component labels should still be interpreted with caution. Following the EchoNext label definitions, a structured field that was not completed was treated as absence of disease, an assumption that carries little weight for the nine labels graded in most studies. For aortic regurgitation and pericardial effusion, graded in a minority, the estimate from all studies and the estimate restricted to graded studies differ appreciably and in opposite directions, the first inflated by ungraded studies the model scores low and the second deflated by the clinical indication that prompted grading. Both are best read as bounds. The convention was validated in the original report where these fields were almost always completed, and that evidence does not extend to a database in which they usually are not.

Subgroup analyses did not identify major degradation by sex or reported race or ethnicity, but they should be read as descriptive performance checks, not evidence of equitable deployment. Discrimination fell with increasing age and was lower in inpatient and intensive care settings, in both cases alongside higher SHD prevalence. Prevalence alone does not account for this, since discrimination is a rank-based measure; a difference in case mix is the more likely explanation, and a similar age gradient is reported for the original model,^9^ suggesting that it reflects the prediction task rather than this database. Race and ethnicity in MIMIC-IV derive from administrative records rather than cardiac metadata, some values were Unknown, and subgroup sizes varied; prospective implementation studies would be needed to assess downstream equity in referral, diagnosis, and treatment.

Clinically, these results position EchoNext-Mini as a candidate risk-stratification and prioritization tool rather than a stand-alone diagnostic test. Any future workflow would require local waveform preprocessing validation, monitoring of label prevalence and calibration, and threshold selection tied to echocardiography capacity and downstream care pathways.^12^

### Limitations

This study has limitations. MIMIC-IV is a single health-system public database, and the cohort comprised patients with clinically obtained ECGs and subsequent echocardiograms rather than an unselected screening population, so the findings describe transportability in a clinically selected setting. Echocardiographic labels were derived from structured fields rather than source reports or images, and some EchoNext variables required harmonized approximations, including the posterior-wall component of LV wall thickness and a reconstructed PASP; atrial rate is not recorded in MIMIC-IV-ECG and was assigned the value specified by the released preprocessing rule for all records, and substituting median imputation left discrimination unchanged for every label (see Supplementary material online, *Table S8*). For aortic regurgitation and pericardial effusion the reference standard was therefore incomplete for most patients.

The study evaluated transportability, recalibration and prioritization, not clinical implementation, patient outcomes, or downstream referral behaviour. Prioritization was assessed by reordering a cohort retrospectively against a random ordering of the same patients, because MIMIC-IV does not record the sequence in which echocardiograms were requested; clinical referral is already prioritized, so the gain over current practice is smaller than that comparison implies. Formal assessment of clinical utility, such as net benefit for echocardiography referral, would require prospective evaluation in an unselected population.^12^ Finally, although the cohort spanned outpatient, emergency, inpatient, and intensive care settings, care setting was assigned by timestamp linkage and the cohort remains enriched for clinical suspicion of cardiovascular disease.

## Conclusion

EchoNext-Mini showed moderate-to-good transportability in MIMIC-IV, with preserved discrimination for the composite SHD label and near-baseline calibration of the composite output. Component-level probabilities were less well calibrated, and this reflected a property of the released model rather than of the validation setting, being equally present on the benchmark test set distributed with it. An adjustment derived from the released training-split prevalences corrected this without local echocardiographic data and left discrimination unchanged, so released models of this kind may be used to prioritize imaging much as distributed, provided component probabilities are adjusted before they are interpreted as absolute risk estimates. These findings support independent validation of the preprocessing pipeline, label mapping, calibration, and subgroup performance before released AI-ECG models are considered for clinical use in new health-system environments.

## Supplementary Material

ztag147_Supplementary_Data

## Data Availability

The data underlying this article are available to credentialled users through PhysioNet under a Data Use Agreement: MIMIC-IV,^14,15^ MIMIC-IV-ECG,^16^ MIMIC-IV-ED,^17^ and MIMIC-IV-ECHO.^18^ Patient-level data are not redistributed by the authors. The EchoNext-Mini model and benchmark dataset are publicly available from the original investigators.^10^ Analysis code, SQL label and cohort definitions, and aggregate results are available at https://github.com/nuel-otabormd/echonext-mimic-validation (version 2.0.5, archived at Zenodo, DOI: 10.5281/zenodo.21894093; all versions: 10.5281/zenodo.20962994).
