# Supplementary material for: Transportability of an artificial intelligence electrocardiography model for structural heart disease: external validation and recalibration of EchoNext-Mini in MIMIC-IV
Source: Eur Heart J Digit Health. 2026 Sep 19;7(8):ztag147. doi: 10.1093/ehjdh/ztag147 (PMC13617667; doi:10.1093/ehjdh/ztag147)
Supplement: Supplementary file 1 [file ztag147_supplementary_data.docx]

**Supplementary Material**

| **#** | **Condition** | **EchoNext definition** | **MIMIC primary definition** | **Sensitivity analyses** | **Prevalence % (MIMIC / EchoNext-Mini)³** |
| --- | --- | --- | --- | --- | --- |
| 1 | Reduced LVEF | numeric LVEF ≤45% | best-available quantitative LVEF (biplane to 3D to visual-range midpoint) ≤45% | midpoint-only; lower-bound | 16.0% / 18.0% |
| 2 | LV wall thickness | max(IVS, posterior wall) ≥1.3 cm | max(septal_thickness, inferolateral wall) ≥1.3 cm¹ | septal thickness ≥1.5 cm; categorical moderate-or-severe LVH descriptors | 22.4% / 18.8% |
| 3 | Aortic stenosis | moderate-or-greater | Graded severity where populated (11.5% of studies); otherwise peak velocity ≥3.0 m/s, mean gradient ≥20 mmHg, or valve area ≤1.5 cm² | graded severity field only | 5.1% / 5.4% |
| 4 | Aortic regurgitation | moderate-or-greater | *aortic_regurg* ≥ moderate (mild-mod = negative) | None | 1.5% / 1.2% |
| 5 | Mitral regurgitation | moderate-or-greater | *mitral_regurg* ≥ moderate (mild-mod = negative) | None | 8.8% / 6.5% |
| 6 | Tricuspid regurgitation | moderate-or-greater | *tricuspid_regurg* ≥ moderate (incl. torrential) | None | 10.3% / 7.0% |
| 7 | Pulmonic regurgitation | moderate-or-greater | pulm_regurg: moderate-severe, severe, or significant | None | 1.6% / 0.4% |
| 8 | RV systolic dysfunction | categorical moderate/severe | categorical rv_function moderate/severe (incl. "depressed") | categorical descriptors plus TAPSE <1.7 cm (sensitivity analysis) | 5.8% / 8.0% |
| 9 | Pericardial effusion | moderate or large | moderate / moderate-large / large (small & below = negative) | tamponade descriptors | 0.6% / 1.3% |
| 10 | Elevated PASP | numeric PASP ≥45 mmHg | TR gradient + IVC-derived RAP ≥45² | fixed RAP 3/5/10 mmHg | 10.8% / 12.9% |
| 11 | Elevated TR Vmax | direct TR Vmax ≥3.2 m/s | direct tr_velocity ≥3.2 m/s | derived gradient *tr_mmhg*≥40.96 | 12.7% / 6.5% |
| 12 | Any SHD (composite) | any of 1–11 | GREATEST(1–11); model output index 11 | None | 47.2% / 43.3% |

**Supplementary Table S1. Label definitions (EchoNext to MIMIC-IV harmonization)**

Labels were derived from MIMIC-IV-ECHO structured transthoracic echocardiographic fields, not from DICOM images or free-text reports. Original EchoNext thresholds were retained wherever the corresponding structured field was available.

MIMIC-IV-ECHO reports septal and inferolateral left ventricular wall thickness but no distinct posterior-wall field, and provides no single pulmonary artery systolic pressure field; right atrial pressure was estimated from the inferior vena cava. Aortic stenosis was taken from the graded severity field where populated and otherwise from guideline quantitative thresholds (peak velocity at least 3.0 m/s, mean gradient at least 20 mmHg, or valve area no greater than 1.5 cm sq).

| **Label** | **MIMIC-IV-ECHO source field** | **Field populated, n (%)** | **Field absent, treated as negative, n (%)** | **Prevalence under this convention, %** |
| --- | --- | --- | --- | --- |
| Reduced LVEF (<=45%) | lvef / biplane_lvef / lvef_3d | 45,878 (100.0) | 0 (0.0) | 16.03 |
| LV wall thickness (>=1.3 cm) | ivs_thickness, inferolateral_thickness | 43,528 (94.9) | 2,350 (5.1) | 22.36 |
| Aortic stenosis | aortic valve severity; av_pk_vel, av_mean_grad, av_area | 42,441 (92.5) | 3,437 (7.5) | 5.07 |
| Aortic regurgitation | aortic regurgitation severity | 15,009 (32.7) | 30,869 (67.3) | 1.54 |
| Mitral regurgitation | mitral regurgitation severity | 42,455 (92.5) | 3,423 (7.5) | 8.83 |
| Tricuspid regurgitation | tricuspid regurgitation severity | 42,754 (93.2) | 3,124 (6.8) | 10.32 |
| Pulmonic regurgitation | pulmonic regurgitation severity | 30,490 (66.5) | 15,388 (33.5) | 1.57 |
| RV dysfunction | RV systolic function descriptor | 44,665 (97.4) | 1,213 (2.6) | 5.78 |
| Pericardial effusion | pericardial effusion size | 8,246 (18.0) | 37,632 (82.0) | 0.59 |
| Elevated PASP (>=45 mmHg) | TR gradient + IVC-estimated RAP | 34,541 (75.3) | 11,337 (24.7) | 10.76 |
| Elevated TR Vmax (>=3.2 m/s) | TR peak velocity | 34,542 (75.3) | 11,336 (24.7) | 12.70 |

**Supplementary Table S2. Per-label structured-field availability and the missing-as-negative convention**

For each label: the MIMIC-IV-ECHO source field, the number of the 45,878 primary-cohort studies in which it was populated, and the resulting prevalence. Structured reports populate qualitative valve and pericardial fields chiefly when a finding is present, so absence of a field is not equivalent to absence of disease; Supplementary Table S7 bounds the consequence for the two labels where it matters.

Ejection fraction is present in 100% of studies by the inclusion criterion.

| **Component** | **Item** | **Maximum absolute difference** | **Mean predicted probability** |
| --- | --- | --- | --- |
| Tabular feature | Sex | 0.000e+00 | - |
| Tabular feature | Age | 0.000e+00 | - |
| Tabular feature | Ventricular rate | 0.000e+00 | - |
| Tabular feature | Atrial rate | 0.000e+00 | - |
| Tabular feature | PR interval | 0.000e+00 | - |
| Tabular feature | QRS duration | 0.000e+00 | - |
| Tabular feature | Corrected QT | 0.000e+00 | - |
| Model output | Reduced LVEF (<=45%) | 0.000e+00 | 0.5104 |
| Model output | LV wall thickness (>=1.3 cm) | 0.000e+00 | 0.4787 |
| Model output | Aortic stenosis | 0.000e+00 | 0.4333 |
| Model output | Aortic regurgitation | 0.000e+00 | 0.4421 |
| Model output | Mitral regurgitation | 0.000e+00 | 0.4716 |
| Model output | Tricuspid regurgitation | 0.000e+00 | 0.4803 |
| Model output | Pulmonic regurgitation | 0.000e+00 | 0.3873 |
| Model output | RV dysfunction | 0.000e+00 | 0.4804 |
| Model output | Pericardial effusion | 0.000e+00 | 0.4588 |
| Model output | Elevated PASP (>=45 mmHg) | 0.000e+00 | 0.4786 |
| Model output | Elevated TR Vmax (>=3.2 m/s) | 0.000e+00 | 0.4703 |
| Model output | Structural heart disease | 0.000e+00 | 0.5434 |

**Supplementary Table S3. Implementation equivalence against the released module**

Feature construction and inference were compared against the official released module on a test set stratified to include records with unmeasurable PR interval, unmeasurable atrial rate, missing QRS duration or corrected QT, and extreme measurement values (n = 39). Maximum absolute difference was zero for every tabular feature and every output, so no result in this study is attributable to a difference in implementation. Mean predicted probability is shown for the outputs only.

| **Label** | **Prevalence, %** | **Mean predicted probability** | **AUROC** | **AUPRC** | **CIL** | **Calibration slope** | **Brier** | **Brier skill** | **CIL after prior shift** | **Brier skill after prior shift** |
| --- | --- | --- | --- | --- | --- | --- | --- | --- | --- | --- |
| Reduced LVEF (<=45%) | 17.68 | 0.354 | 0.852 | 0.599 | -0.177 | 1.12 | 0.145 | +0.003 | -0.014 | +0.290 |
| LV wall thickness (>=1.3 cm) | 19.50 | 0.401 | 0.734 | 0.372 | -0.206 | 0.99 | 0.188 | -0.198 | -0.008 | +0.106 |
| Aortic stenosis | 5.26 | 0.343 | 0.859 | 0.248 | -0.291 | 1.34 | 0.161 | -2.225 | +0.018 | +0.092 |
| Aortic regurgitation | 1.21 | 0.340 | 0.740 | 0.032 | -0.328 | 0.77 | 0.171 | -13.256 | +0.002 | +0.009 |
| Mitral regurgitation | 6.19 | 0.341 | 0.806 | 0.222 | -0.279 | 0.94 | 0.166 | -1.849 | -0.011 | +0.091 |
| Tricuspid regurgitation | 6.49 | 0.349 | 0.833 | 0.292 | -0.284 | 1.12 | 0.157 | -1.587 | -0.023 | +0.122 |
| Pulmonic regurgitation | 0.37 | 0.258 | 0.832 | 0.116 | -0.254 | 1.00 | 0.128 | -34.086 | -0.002 | +0.035 |
| RV dysfunction | 7.70 | 0.329 | 0.866 | 0.428 | -0.252 | 1.15 | 0.145 | -1.034 | -0.031 | +0.203 |
| Pericardial effusion | 1.27 | 0.400 | 0.766 | 0.072 | -0.387 | 1.25 | 0.190 | -14.186 | -0.013 | +0.006 |
| Elevated PASP (>=45 mmHg) | 12.84 | 0.379 | 0.770 | 0.357 | -0.251 | 1.00 | 0.174 | -0.553 | -0.027 | +0.120 |
| Elevated TR Vmax (>=3.2 m/s) | 6.89 | 0.363 | 0.754 | 0.211 | -0.295 | 0.89 | 0.174 | -1.718 | -0.016 | +0.057 |
| Structural heart disease | 42.59 | 0.412 | 0.820 | 0.789 | +0.014 | 1.03 | 0.169 | +0.309 | -0.002 | +0.309 |

**Supplementary Table S4. Performance on the released benchmark test set, before and after the prior-shift correction**

Inputs were the distributed arrays, used without modification, so no preprocessing performed for this study contributed to these values. Areas under the receiver operating characteristic curve reproduce the published values to three decimal places for 10 of 12 labels; the exceptions are aortic regurgitation (0.740 against 0.739) and pulmonic regurgitation (0.832 against 0.829), the two labels with fewest events in this test set. Mean predicted probability lies between 0.26 and 0.40 for all component labels despite prevalence of 0.4%–19.5%, and Brier skill is negative for 10 of 11 components and positive for the composite. The prior shift uses only the released training-split prevalences and no outcome data from this dataset.

| **Subgroup** | **n** | **Prevalence, %** | **AUROC (95% CI)** | **Calibration slope** | **CIL** | **Brier skill** |
| --- | --- | --- | --- | --- | --- | --- |
| **Sex** |  |  |  |  |  |  |
| Male | 23,569 | 51.4 | 0.778 (0.772–0.784) | 0.89 | +0.043 | +0.222 |
| Female | 22,309 | 42.7 | 0.801 (0.795–0.806) | 0.97 | +0.011 | +0.266 |
| **Age** |  |  |  |  |  |  |
| Under 65 years | 19,492 | 31.7 | 0.773 (0.766–0.779) | 0.91 | -0.009 | +0.213 |
| 65–79 years | 15,360 | 50.9 | 0.751 (0.743–0.758) | 0.84 | +0.034 | +0.181 |
| 80 years and older | 11,026 | 69.5 | 0.732 (0.722–0.742) | 0.79 | +0.083 | +0.091 |
| **Race or ethnicity** |  |  |  |  |  |  |
| White | 31,775 | 48.3 | 0.786 (0.781–0.791) | 0.93 | +0.024 | +0.241 |
| Black | 6,053 | 47.9 | 0.802 (0.791–0.812) | 0.97 | +0.063 | +0.258 |
| Hispanic | 2,116 | 37.9 | 0.778 (0.758–0.799) | 0.89 | +0.022 | +0.228 |
| Asian | 1,486 | 38.4 | 0.806 (0.783–0.830) | 0.98 | -0.005 | +0.279 |
| Other | 1,706 | 40.7 | 0.802 (0.781–0.823) | 0.97 | +0.001 | +0.273 |
| Unknown | 2,742 | 49.1 | 0.798 (0.781–0.814) | 0.94 | +0.032 | +0.259 |
| **Care setting at ECG (detailed)** |  |  |  |  |  |  |
| Emergency or acute presentation | 13,191 | 44.4 | 0.785 (0.778–0.793) | 0.93 | +0.017 | +0.244 |
| Intensive care | 7,160 | 61.0 | 0.741 (0.729–0.752) | 0.81 | +0.046 | +0.150 |
| Inpatient ward | 14,332 | 50.9 | 0.783 (0.775–0.790) | 0.93 | +0.025 | +0.236 |
| Outpatient or ambulatory | 11,195 | 36.9 | 0.796 (0.787–0.805) | 0.99 | +0.033 | +0.256 |
| **Care setting at ECG (grouped)** |  |  |  |  |  |  |
| Emergency or acute presentation | 13,191 | 44.4 | 0.785 (0.778–0.793) | 0.93 | +0.017 | +0.244 |
| Inpatient or intensive care | 21,492 | 54.3 | 0.773 (0.767–0.780) | 0.90 | +0.032 | +0.216 |
| Outpatient or ambulatory | 11,195 | 36.9 | 0.796 (0.787–0.805) | 0.99 | +0.033 | +0.256 |
| **ECG acquisition setting (bandwidth, filtering)** |  |  |  |  |  |  |
| 0.0005–150 Hz, 60 Hz notch Baseline filter | 236 | 63.1 | 0.768 (0.707–0.829) | 1.00 | +0.041 | +0.194 |
| 0.0005–150 Hz, <not specified> | 6,256 | 46.7 | 0.773 (0.762–0.785) | 0.88 | +0.026 | +0.222 |
| 0.005–150 Hz, 50 Hz notch Baseline filter | 190 | 59.5 | 0.793 (0.726–0.854) | 1.05 | +0.054 | +0.233 |
| 0.005–150 Hz, 60 Hz notch Baseline filter | 35,763 | 47.7 | 0.792 (0.788–0.797) | 0.94 | +0.031 | +0.251 |
| 0.005–150 Hz, Baseline filter | 541 | 42.0 | 0.845 (0.811–0.877) | 1.23 | -0.010 | +0.360 |
| 0.05–150 Hz, 60 Hz notch Baseline filter | 2,840 | 41.4 | 0.790 (0.773–0.807) | 0.91 | +0.000 | +0.249 |
| **PR interval availability** |  |  |  |  |  |  |
| PR interval measurable | 38,852 | 42.7 | 0.776 (0.772–0.781) | 0.91 | +0.028 | +0.225 |
| PR interval unmeasurable | 7,026 | 72.2 | 0.737 (0.725–0.749) | 1.02 | +0.025 | +0.137 |

**Supplementary Table S5. Composite performance by subgroup, including ECG acquisition setting and PR-interval availability**

Subgroups with fewer than 100 patients or 20 events were not evaluated. Acquisition setting combines the recording bandwidth and filtering reported in the ECG header. CIL; calibration-in-the-large

| **Echocardiographic capacity, % of queue** | **Studies performed** | **Cases identified, model-ranked, %** | **Cases identified, unprioritized, %** | **Enrichment** | **Positive predictive value of studies performed, %** |
| --- | --- | --- | --- | --- | --- |
| 1.0 | 459 | 2.0 | 1.0 | 2.02 | 95.4 |
| 2.5 | 1,147 | 5.0 | 2.5 | 2.00 | 94.3 |
| 5.0 | 2,294 | 9.8 | 5.0 | 1.97 | 92.9 |
| 10.0 | 4,588 | 19.1 | 10.0 | 1.91 | 90.1 |
| 20.0 | 9,176 | 35.7 | 20.0 | 1.79 | 84.3 |
| 30.0 | 13,763 | 50.0 | 30.0 | 1.67 | 78.8 |
| 50.0 | 22,939 | 73.0 | 50.0 | 1.46 | 68.9 |

**Supplementary Table S6. Prioritization of echocardiography under fixed capacity**

The unprioritized comparator is a random ordering of the same cohort, because MIMIC-IV does not record a referral sequence. At the observed prevalence of 47.2% the maximum achievable enrichment at low capacity is 2.12.

| **Label** | **Source field populated, %** | **Prevalence all records, %** | **AUROC all records** | **n complete-case** | **Prevalence complete-case, %** | **AUROC complete-case** | **Difference** |
| --- | --- | --- | --- | --- | --- | --- | --- |
| Reduced LVEF (<=45%) | 100.0 | 16.03 | 0.835 | 45,878 | 16.03 | 0.835 | +0.000 |
| LV wall thickness (>=1.3 cm) | 94.9 | 22.36 | 0.679 | 43,528 | 23.57 | 0.686 | +0.007 |
| Aortic stenosis | 92.5 | 5.07 | 0.783 | 42,441 | 5.48 | 0.782 | -0.001 |
| Aortic regurgitation | 32.7 | 1.54 | 0.730 | 15,009 | 4.70 | 0.634 | -0.096 |
| Mitral regurgitation | 92.5 | 8.83 | 0.806 | 42,455 | 9.54 | 0.806 | -0.000 |
| Tricuspid regurgitation | 93.2 | 10.32 | 0.805 | 42,754 | 11.07 | 0.808 | +0.003 |
| Pulmonic regurgitation | 66.5 | 1.57 | 0.723 | 30,490 | 2.36 | 0.740 | +0.016 |
| RV dysfunction | 97.4 | 5.78 | 0.837 | 44,665 | 5.94 | 0.839 | +0.002 |
| Pericardial effusion | 18.0 | 0.59 | 0.765 | 8,246 | 3.27 | 0.680 | -0.086 |
| Elevated PASP (>=45 mmHg) | 75.3 | 10.76 | 0.768 | 34,541 | 14.29 | 0.760 | -0.008 |
| Elevated TR Vmax (>=3.2 m/s) | 75.3 | 12.70 | 0.763 | 34,542 | 16.87 | 0.757 | -0.006 |

**Supplementary Table S7. Complete-case restriction for labels with incomplete field availability**

The all-studies estimate is inflated by ungraded studies that the model scores low; the complete-case estimate is deflated because fields are populated by indication. The two are bounds, and neither is unbiased.

| **Analysis** | **Prevalence, %** | **AUROC** | **CIL** | **Calibration slope** | **Brier skill** |
| --- | --- | --- | --- | --- | --- |
| RV dysfunction: categorical only (PRIMARY) | 5.78 | 0.837 | -0.298 | 1.11 | -2.076 |
| RV dysfunction: categorical + TAPSE | 6.03 | 0.832 | -0.295 | 1.09 | -1.959 |
| TR Vmax: direct velocity (PRIMARY) | 12.70 | 0.763 | -0.264 | 1.04 | -0.646 |
| TR Vmax: derived gradient | 11.39 | 0.766 | -0.277 | 1.05 | -0.816 |
| Aortic stenosis: rebuilt (PRIMARY) | 5.07 | 0.783 | -0.354 | 0.94 | -3.464 |
| Aortic stenosis: graded severity field only | 4.46 | 0.781 | -0.360 | 0.93 | -4.078 |
| LVWT: max(septal, inferolateral) (PRIMARY) | 22.36 | 0.679 | -0.195 | 0.79 | -0.191 |
| LVWT: septal >=1.5 cm | 4.00 | 0.746 | -0.379 | 1.22 | -4.336 |
| LVWT: categorical moderate-or-severe | 3.24 | 0.754 | -0.387 | 1.28 | -5.562 |
| PASP: TR gradient + IVC RAP (PRIMARY) | 10.76 | 0.768 | -0.294 | 1.09 | -0.961 |
| PASP: phtn_severity moderate-or-severe | 16.18 | 0.764 | -0.240 | 1.07 | -0.358 |
| Composite: rebuilt AS (PRIMARY) | 47.21 | 0.790 | +0.028 | 0.93 | +0.249 |
| Composite: graded severity field only | 47.06 | 0.790 | +0.026 | 0.93 | +0.249 |
| ECG-to-echocardiogram window <=30d | 48.72 | 0.789 | +0.026 | 0.93 | +0.247 |
| ECG-to-echocardiogram window <=90d | 47.54 | 0.791 | +0.023 | 0.94 | +0.252 |
| ECG-to-echocardiogram window <=180d | 47.18 | 0.792 | +0.024 | 0.94 | +0.252 |
| ECG-to-echocardiogram window <=365d | 47.21 | 0.790 | +0.028 | 0.93 | +0.249 |
| RV dysfunction excluding unassessable studies | 5.93 | 0.839 | -0.295 | 1.12 | -1.980 |
| Atrial rate filled with zero, as released (PRIMARY) | 47.21 | 0.790 | +0.028 | 0.93 | +0.249 |
| Atrial rate median-imputed | 47.21 | 0.790 | +0.014 | 0.93 | +0.251 |

**Supplementary Table S8. Sensitivity analyses across alternative label definitions, linkage windows and input-handling rules**

All analyses use the same single label source as the primary analysis. All performance metrics are reported as point estimates without confidence intervals.

| **Label definition** | **Prevalence, %** | **AUROC** | **Difference versus EchoNext definition** |
| --- | --- | --- | --- |
| Maximum of septum and posterior wall (EchoNext definition) | 22.02 | 0.712 | +0.000 |
| Interventricular septum alone | 19.50 | 0.713 | +0.002 |
| Posterior wall alone | 13.06 | 0.725 | +0.014 |

**Supplementary Table S9. Effect of restricting the wall-thickness label to a single wall, in the released benchmark test set**

Restricted to the 4,810 of 5,442 test records in which both walls were measured (88.4%). The published label flag agrees with a threshold of 1.3 cm applied to the maximum of the two walls in 100.0% of these records. MIMIC-IV-ECHO records septal and inferolateral wall thickness but no posterior wall measurement, so the substitution used in this study could not be evaluated directly; omitting one wall entirely, a more severe perturbation than substituting an anatomically adjacent one, is shown here as a bound.

**Supplementary Figure S1. Reliability curves for the remaining component labels.**

Observed frequency against predicted probability in equal-sized bins for the eight components not shown in Figure 3, as released and after the prior-shift correction. Both axes are logarithmic and are shared across all eight panels, which keeps the low-prevalence labels legible and makes the panels directly comparable. The dotted line marks each label's observed prevalence.

*Alt text: Eight panels plotting observed frequency against predicted probability on logarithmic axes against a diagonal reference line, the released-model curve away from the diagonal and the corrected curve close to it.*

**Supplementary Figure S2. Composite performance by ECG acquisition setting.**

Composite AUROC with 95% confidence intervals for each combination of recording bandwidth and filtering, with the overall cohort estimate shown as a dashed reference.

*Alt text: Horizontal forest plot of composite AUROC by acquisition setting, point size proportional to the number of patients, against a dashed vertical reference at the overall estimate.*

**Supplementary Figure S3. Final-layer bias against the log-odds of training prevalence.**

Each point is one of the twelve outputs. In panel A a model emitting probabilities on the natural prevalence scale would lie on the identity line, and the axes share a common range so that line is a true diagonal. Panel B shows the same twelve values on an axis scaled to the biases themselves, each label given with its prevalence in the training split.

*Alt text: Two panels. Panel A is a scatter plot of final-layer bias against log-odds of training prevalence with an identity line, all twelve points lying near zero on the vertical axis while spread widely on the horizontal axis. Panel B is a dot plot of the same twelve biases on an expanded axis, every one within 0.05 of zero.*

| **#** | **Item** | **Addressed** | **Where** |
| --- | --- | --- | --- |
| 1 | Title identifies study as AI model evaluation | Yes | Title |
| 2 | Structured abstract | Yes | Abstract |
| 3 | Background/rationale, intended use, role of AI | Yes | Introduction |
| 4 | Objectives | Yes | Introduction |
| 5 | Data source & study design | Yes | Methods (Data source and cohort) |
| 6 | Eligibility / inclusion–exclusion | Yes | Methods (Cohort); Supplementary Table S1 |
| 7 | Outcome (label) definition & assessment | Yes | Methods (Label harmonization); Supplementary Table S1 |
| 8 | Predictors / model inputs | Yes | Methods (Model and preprocessing) |
| 9 | Missing data handling | Yes | Methods (Statistical analysis); Supplementary Table S2 |
| 10 | Model (released EchoNext-Mini); architecture; version | Yes | Methods (Model and preprocessing); released model used without retraining |
| 11 | Model output & post-processing (sigmoid probabilities) | Yes | Methods (Model and preprocessing) |
| 12 | Analysis: discrimination, calibration, recalibration, utility | Yes | Methods (Statistical analysis); Results |
| 13 | Subgroup/fairness analysis | Yes | Methods (Statistical analysis); Results (Subgroup performance) |
| 14 | Sample size / events | Yes | Results; Table 1 |
| 15 | Participants flow (attrition) | Yes | Figure 1 |
| 16 | Participant characteristics (Table 1) | Yes | Table 1 |
| 17 | Model performance (discrimination + CIs) | Yes | Table 2 |
| 18 | Calibration + recalibration results | Yes | Results; Figure 3; Supplementary Figure S1; Supplementary Table S4 |
| 19 | Clinical utility | Yes | Results (Prioritization of echocardiography); Figure 4; Supplementary Table S6. Decision-curve net benefit is reported; utility in an unselected population would still require prospective evaluation. |
| 20 | Fairness results | Yes | Results (Subgroup performance); Table 5; Supplementary Table S5 |
| 21 | Sensitivity analyses | Yes | Results (Sensitivity analyses); Supplementary Table S8 |
| 22 | Interpretation | Yes | Discussion |
| 23 | Limitations | Yes | Discussion |
| 24 | Fairness/equity implications | Yes | Discussion |
| 25 | Data availability | Yes | Data Availability statement |
| 26 | Code availability | Yes | Data Availability statement |
| 27 | Funding / conflicts / AI use | Yes | Funding; Conflicts of Interest; Methods (AI-assisted tools) |
| 28 | Reproducibility (env, seeds, deterministic cohort) | Yes | Data Availability statement (public repository; deterministic cohort and analysis code) |

**TRIPOD+AI Reporting Checklist**

Mapping of TRIPOD+AI items to where each is addressed in the manuscript and supplementary material.
